# Meta-analytic prospective associations between self-esteem and eating disorders appear to be spurious: a reanalysis and comment on Krauss et al. (2023)

**DOI:** 10.3389/fpsyg.2025.1463701

**Published:** 2025-02-07

**Authors:** Kimmo Sorjonen, Ata Ghaderi, Bo Melin

**Affiliations:** Karolinska Institutet (KI), Solna, Sweden

**Keywords:** cross-lagged effects, eating disorders, meta-analysis, overinterpretation of findings, reanalysis, self-esteem, spurious associations

## Abstract

In a recent meta-analysis, Krauss et al. found support for a reciprocal model of low self-esteem and eating disorders where, in a vicious circle, low self-esteem makes people more vulnerable to developing eating disorders and eating disorders, in turn, scars individuals’ self-esteem. However, in the present reanalyses of the same meta-analytic data, we found that the prospective effects between self-esteem and eating disorders are likely spurious, meaning they do not reflect a true causal effect, but rather correlations with residuals and regression to the mean. Consequently, the claims by Krauss et al. can be challenged. To avoid statistical artifacts, we recommend researchers to fit, as we did in the present study, complementary models to their data in order to evaluate if prospective effects may be genuinely increasing or decreasing or if they appear to be spurious.

## Introduction

Self-esteem is often defined as our attitudes toward ourselves, where high and low self-esteem would mean a positive and a negative attitude, respectively (Kenrick et al., 2007). It has been proposed that low self-esteem increases the likelihood for eating pathology (Colmsee et al., 2021). It has also been hypothesized that eating pathology may, conversely, have a detrimental impact on self-esteem (Silverstone and Salsali, 2003).

Krauss et al. (2023) extracted zero-order correlations between self-esteem and eating disorders measured at two occasions from studies included in their meta-analysis and estimated the effect of prior self-esteem on subsequent eating disorders when adjusting for prior eating disorders and vice versa. As both meta-analytic adjusted cross-lagged effects were statistically significant (*β* = −0.08 and *β* = −0.09, respectively), Krauss et al. concluded that their findings supported a reciprocal-relations model of low self-esteem and eating disorders where, in a vicious circle, low self-esteem makes people more vulnerable to developing eating disorders and eating disorders, in turn, deteriorates individuals’ self-esteem. However, it is well established that adjusted cross-lagged effects may be spurious due to correlations with residuals and regression to the mean rather than due to genuine increasing or decreasing effects (Glymour et al., 2005; Eriksson and Häggström, 2014; Castro-Schilo and Grimm, 2018; Sorjonen et al., 2019; Lucas, 2023).

For example, self-rated self-esteem and eating pathology are negatively correlated (Krauss et al., 2023). Therefore, we should expect that among individuals with the same initial degree of eating pathology, those with a higher initial self-esteem score have a lower true degree of eating pathology and have, consequently, received a more positive residual in the measurement of eating pathology compared with those with the same initial measured eating pathology score but lower initial self-esteem. However, residuals tend to regress toward a mean value of zero between measurements. This means that we should expect a more negative, but spurious, change in self-rated eating pathology to a subsequent measurement among those with high initial self-esteem compared with those with the same initial eating pathology score but lower initial self-esteem. This combination of correlations with residuals and regression toward the mean might explain observed negative cross-lagged effects of initial self-esteem on subsequent eating pathology when adjusting for initial eating pathology and vice versa.

The objective of the present study was to reanalyze the meta-analytic data used by Krauss et al. (2023) in order to evaluate if the identified adjusted cross-lagged associations suggested genuine decreasing effects between self-esteem and eating disorders or if the associations may have been spurious.

## Method

### Meta-analytic data

We refer to Krauss et al. (2023) for more comprehensive information on selection of studies, study populations, etc. In short, Krauss et al. extracted information from 44 sources (41 journal articles and three dissertations, published between 2001 and 2018) on 48 independent samples (ranging in size between 20 and 2,601, *M* = 399.7, total *N* = 19,187). In total, the data provided between 127 and 146 correlations between self-esteem and eating pathology measured at two occasions. Mean age at the initial measurement ranged between 6.5 years and 47.6 years (*M* = 19.3 years). Mean proportion of female participants was 79% (range from 0 to 100%). The two most commonly used measures of self-esteem and eating disorders were Rosenberg Self-Esteem Scale, Harter’s Self-Perception Profile, Eating Disorder Examination, and Eating Disorder Inventory, respectively. Additionally, an Italian tool for measuring eating behavior regulation, the Tempest Self-Regulation Questionnaire for Eating, has been validated in the literature, showing adequate psychometric properties and gender invariance (Diotaiuti et al., 2022). Krauss et al. have made their data and analytic script available at https://osf.io/t6nge/.

### Statistical analyses and predictions

**Figure 1:**
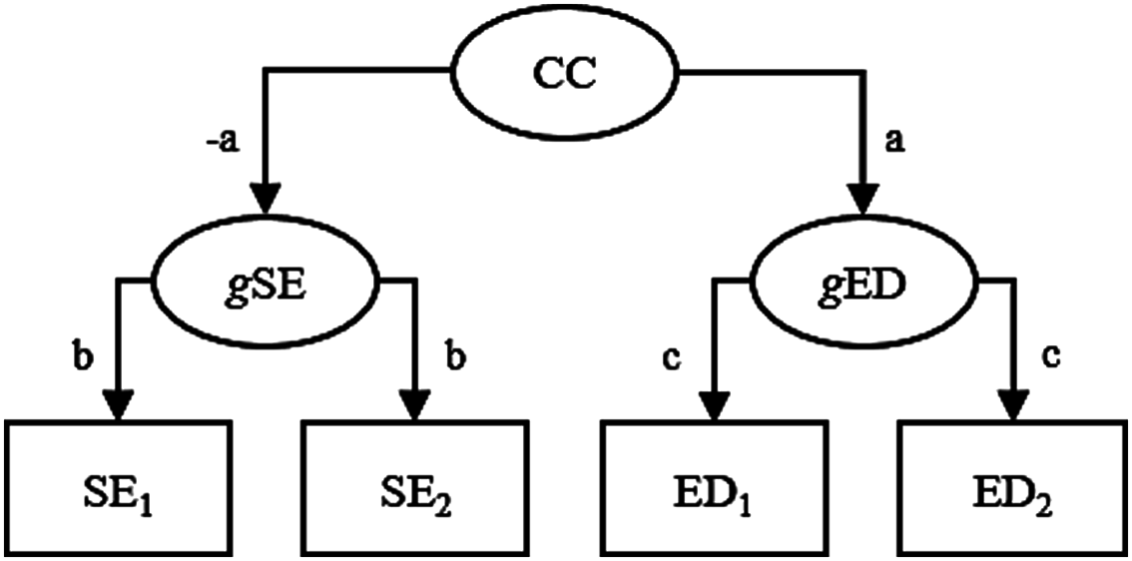
Hypothetical data generating model without genuine direct effects between self-esteem and eating disorder. Here, measured self-esteem and eating disorders at two occasions are affected by general levels of self-esteem and eating disorders, respectively, and these general levels are, in turn, affected by some common (confounding) factors (with opposite signs of the effects). These common factors could, for example, be various personality (e.g., body dissatisfaction) and environmental factors. With parameter-values between (but not including) 0 and 1 for *a, b,* and *c*, the model predicts negative cross-lagged effects of SE_1_ on ED_2_ when adjusting for ED_1_ and vice versa, negative effects of SE_1_ on ED_1_ when adjusting for ED_2_ and vice versa, and null effects of SE_1_ on the ED_2_-ED_1_ difference and vice versa. CC, common cause; SE_1_, SE_2_, ED_1_, ED_2_, initial and subsequent self-esteem and eating disorder, respectively; *g*SE, general self-esteem; *g*ED, general eating disorder.

Following Krauss et al. (2023), we used Equation 1 (Cohen et al., 2003) to estimate the effect of initial self-esteem on subsequent eating pathology while adjusting for initial eating pathology, and vice versa, in the included studies. Here, both a hypothesis of genuine reciprocal decreasing effects and a hypothesis of spurious effects (if data were, for example, generated as in Figure 1) predicted negative effects (Table 1, rows 1 and 4). Additionally, we used Equation 1 to estimate the effect of initial self-esteem on initial eating pathology while adjusting for subsequent eating pathology and vice versa. Here, a hypothesis of genuine decreasing reciprocal effects predicted positive effects. This positive effect would indicate that a high initial score on self-esteem had counteracted a high initial value on eating pathology and allowed individuals to reach the same subsequent value on eating pathology as individuals with a lower initial value on eating pathology but also with a lower initial value on self-esteem and vice versa. Contrarily, a hypothesis of spurious effects predicted these effects to be negative (Table 1, rows 2 and 5). Moreover, we used Equation 2 (Guilford, 1965) to estimate the effect of initial self-esteem on the subsequent – initial eating pathology difference and vice versa. Here, a hypothesis of genuine reciprocal decreasing effects predicted negative effects. On the other hand, a hypothesis of spurious effects predicted either effects close to zero (if concurrent and cross-lagged correlations were approximately equal in size) or positive effects (if concurrent correlations were more negative than cross-lagged correlations) (Table 1, rows 3 and 6).


(1) $$E|\beta_{X1,Y2.Y1}|\;=\frac{r_{X1,Y2}-r_{X1,Y1}r_{Y1,Y2}}{1-r_{X1,Y1}^{2}}$$

(2) $$E|\beta_{X1,Y2-Y1}|\;=\frac{r_{X1,Y2}-r_{X1,Y1}}{\sqrt{2\left(1-r_{Y1,Y2}\right)}}$$

**Table 1:** Predicted sign of effects between self-esteem and eating disorders according to a hypothesis of genuine reciprocal decreasing effects and a hypothesis of spurious effects.

Effect	Genuine	Spurious
1. β(SE1,ED2.ED1)	Negative	Negative
2. β(SE1,ED1.ED2)	Positive	Negative
3. β(SE1,ED2-ED1)	Negative	Zero or positive
4. β(ED1,SE2.SE1)	Negative	Negative
5. β(ED1,SE1.SE2)	Positive	Negative
6. β(ED1,SE2-SE1)	Negative	Zero or positive

SE, self-esteem; ED, eating disorders; 1, time 1; 2, time 2; the variables are given in the order predictor, outcome, and covariate.

We conducted a multilevel random effects meta-analysis for all six regression effects in Table 1, as well as for each of the six zero-order correlations between self-esteem and eating disorders measured at two occasions. Multilevel analyses adjusted for non-independence due to the fact that some of the effect sizes (*NE* between 127 and 146) were estimated/clustered in the same sample (*K* between 37 and 48). In a first step, pooled effects were estimated across all effects within the same sample, and in a second step an aggregated effect was estimated across all samples. This way, artificially reduced heterogeneity in effect sizes and increased risk for false positive findings were counteracted (Harrer et al., 2022). A random meta-analytic effect, with a 95% confidence interval, was estimated across the independent effect sizes (i.e., independent samples). Analyses were conducted on Fisher’s z-transformed standardized regression effects and correlations, but these were inverted back to non-transformed effects and correlations for presentations. Analyses were conducted with R 4.3.1 statistical software (R Core Team, 2024) using the metafor (Viechtbauer, 2010) and osfr (Wolen et al., 2020) packages. Data, a list of included studies, analytic script, forest plots, and supplementary results are available at the Open Science Framework at https://osf.io/g8dkc/. Similarly as the original meta-analysis by Krauss et al. (2023), the present reanalysis of publicly available, anonymous, and aggregated data did not require ethical approval or consent from participants and it was not pre-registered.

## Results

Meta-analytic estimates of associations between self-esteem and eating disorders are presented in Table 2. With one exception, estimated associations (aggregated within samples) exhibited statistically non-significant and mostly low heterogeneity, as estimated by Cochran’s *Q* and *I^2^*, which estimates percentage of variation across effects attributable to heterogeneity rather than random variance. This means that associations across the included samples can, with one exception, be assumed to have come from the same distribution.

**Table 2:** Meta-analytic correlations and regression effects between self-esteem and eating disorders measured at two occasions.

Association	*K*	*NE*	*N*	Estimate (95% CI)	*Q* (df)	*I*^2^ (95% CI)
1. *r*(SE1,ED1)	48	146	19,187	−0.339 (−0.378; −0.298)	49.2 (47)	0 (0; 41.7)
2. *r*(SE2,ED2)	37	127	15,737	−0.398 (−0.442; −0.352)	39.9 (36)	0 (0; 50.4)
3. *r*(SE1,SE2)	39	133	15,878	0.572 (0.512; 0.626)	38.7 (38)	0 (0; 41.6)
4. *r*(ED1,ED2)	46	140	19,046	0.587 (0.540; 0.630)	44.8 (45)	0 (0; 36.8)
5. *r*(SE1,ED2)	46	140	19,046	−0.266 (−0.300; −0.232)	43.3 (45)	0 (0; 34.5)
6. *r*(ED1,SE2)	39	133	15,878	−0.273 (−0.312; −0.233)	35.8 (38)	0 (0; 35.7)
7. β(SE1,ED2.ED1)	46	140	19,046	−0.079 (−0.096; −0.062)	47.6 (45)	0 (0; 48.9)
8. β(SE1,ED1.ED2)	46	140	19,046	−0.202 (−0.236; −0.167)	44.8 (45)	0 (0; 37.8)
9. β(SE1,ED2-ED1)	46	140	19,046	0.083 (0.054; 0.112)	46.1 (45)	0 (0; 40.8)
10. β(ED1,SE2.SE1)	39	133	15,878	−0.088 (−0.102; −0.075)	56.8 (38)*	0 (0; 84.0)
11. β(ED1,SE1.SE2)	39	133	15,878	−0.202 (−0.243; −0.161)	48.3 (38)	0 (0; 60.7)
12. β(ED1,SE2-SE1)	39	133	15,878	0.068 (0.043; 0.094)	44.5 (38)	0 (0; 57.9)

K, number of samples; NE, number of effects; N, total sample size; Q, Cochran’s *Q*; *I*^2^, percentage of variation due to heterogeneity rather than randomness; SE, self-esteem; ED, eating disorders; 1, time 1; 2, time 2; the variables are given in the order predictor, outcome, and covariate; A hypothesis of genuine decreasing effects predicted the effects on rows 7–12 to be (7) negative, (8) positive, (9) negative, (10) negative, (11) positive, and (12) negative, respectively (see Table 1). As seen, the observed effect on rows 7–12 did not agree with these predictions. However, the observed effects did agree with predictions by a hypothesis of spurious effects of (7) negative, (8) negative, (9) zero or positive, (10) negative, (11) negative, and (12) zero or positive effects, respectively (see Table 1); *p < 0.05.

**Figure 2:**
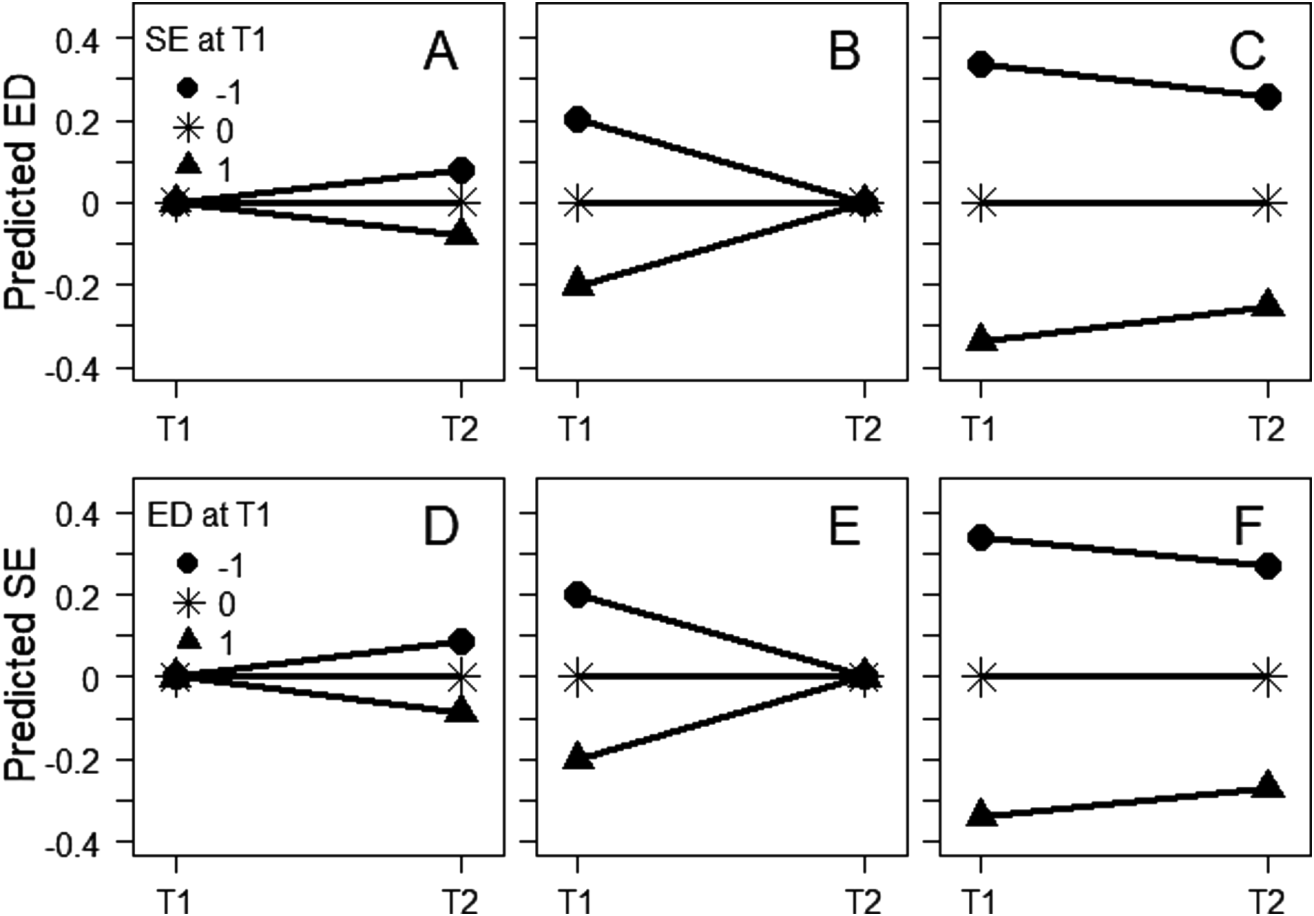
Predicted eating disorders **(A–C)** and self-esteem **(D–F)** at time 1 and time 2, when conditioning on mean initial **(A,D)** and subsequent **(B,E)** score on the outcome variable and when not conditioning **(C,F)**. Separately for individuals with low (*Z* = −1), mean, and high (*Z* = 1) initial self-esteem **(A–C)** and eating disorder **(D–F)**, respectively. The depicted effects of initial self-esteem **(A–C)** and initial eating disorders **(D–F)** across panels **(A–F)** were **(A)** negative, **(B)** negative, **(C)** positive, **(D)** negative, **(E)** negative, and **(F)** positive, respectively. These effects did not agree with predictions by a hypothesis of genuine decreasing effects (see Table 1) but they did agree with predictions by a hypothesis of spurious effects (see Table 1). SE, self-esteem; ED, eating disorder; T1, time 1; T2, time 2.

Concurrent and cross-lagged meta-analytic correlations were negative (Table 2, rows 1–2 and 5–6, respectively) while auto-correlations were positive (Table 2, rows 3–4). As for regression effects, initial self-esteem had a negative effect on subsequent eating pathology when adjusting for initial eating pathology (Table 2, row 7 and Figure 2A) and vice versa (Table 2, row 10 and Figure 2D). Initial self-esteem also had a negative effect on initial eating pathology when adjusting for subsequent eating pathology (Table 2, row 8 and Figure 2B) and vice versa (Table 2, row 11 and Figure 2E). On the other hand, initial self-esteem had a positive effect on the subsequent-initial eating pathology difference (Table 2, row 9 and Figure 2C) and vice versa (Table 2, row 12 and Figure 2F). The regression effects in Table 2, illustrated in Figure 2, indicate that initial self-esteem had a decreasing effect on eating pathology only when conditioning on initial eating pathology, and vice versa. The other effects indicate, contrarily and paradoxically, increasing effects. The negative effect of initial self-esteem on initial eating pathology when adjusting for subsequent eating pathology and vice versa (Table 2, rows 8 and 11 and Figures 2B,E) suggested that low, rather than high, initial self-esteem had counteracted high initial eating pathology and allowed individuals to attain the same subsequent degree of eating pathology as individuals with lower initial eating pathology but with higher initial self-esteem, and vice versa. The meta-analytic regressions effects agreed better with a hypothesis of spurious prospective effects than with a hypothesis of genuine decreasing prospective effects (compare effects on rows 7–12 in Table 2 with predictions in Table 1).

While a hypothesis of genuine decreasing effects predicted the six effects on rows 7–12 in Table 2, and across Figures 2A–F, to be (1) negative, (2) positive, (3) negative, (4) negative, (5) positive, and (6) negative, respectively (see Table 1), the observed effects were (1) negative, (2) negative, (3) positive, (4) negative, (5) negative, and (6) positive, respectively (see rows 7–12 in Table 2 and Figures 2A–F). Consequently, the predictions by a hypothesis of genuine decreasing effects (see Table 1) did not agree with the empirical findings (see rows 7–12 in Table 2 and Figures 2A–F). On the other hand, a hypothesis of spurious effects predicted the six effects on rows 7–12 in Table 2, and across Figures 2A–F, to be (1) negative, (2) negative, (3) zero or positive, (4) negative, (5) negative, and (6) zero or positive, respectively (see Table 1). These predictions by a hypothesis of spurious effects agreed with the observed effects (see rows 7–12 in Table 2 and Figures 2A–F).

The results above are for total eating pathology, i.e., for an aggregation of various eating disorder related variables. Krauss et al. (2023) also analyzed meta-analytic data on specific eating disorder related variables, namely restrained eating, binge eating, bulimia, eating concerns, body dissatisfaction, and drive for thinness. In reanalyses we found mainly a similar pattern of effects for these specific variables as for total eating pathology, i.e., (1) A negative effect of initial self-esteem on subsequent eating pathology when adjusting for initial eating pathology and vice versa (indicating decreasing effects); (2) A negative effect of initial self-esteem on initial eating pathology when adjusting for subsequent eating pathology and vice versa (indicating increasing effects); (3) A positive effect of initial self-esteem on the subsequent-initial eating pathology difference and vice versa (indicating increasing effects) (see Supplementary Tables S1–S6 in results at https://osf.io/g8dkc/).

## Discussion

The objective of the present study was to reanalyze the meta-analytic data used by Krauss et al. (2023) in order to evaluate if the identified adjusted cross-lagged associations suggested genuine decreasing effects between self-esteem and eating disorders or if the associations may have been spurious. The disparate findings of simultaneous and paradoxical decreasing and increasing effects indicated that prospective effects between initial and subsequent self-esteem and eating pathology were spurious, presumably due to correlations with residuals and regression to the mean. Among individuals with the same initial eating disorder score, those with a higher initial self-esteem score may be suspected to have received a too high eating disorder score, i.e., a positive residual, or those with a lower self-esteem score may have received a too low eating disorder score, i.e., a negative residual. However, as residuals tend to regress toward a mean value of zero between measurements, individuals with a higher initial self-esteem score should be expected to experience a more negative, but spurious, change in the eating disorder score between measurements compared with individuals with the same initial eating disorder score but with a lower initial self-esteem score. This might explain the negative effect of initial self-esteem on subsequent eating disorders when adjusting for initial eating disorders. The effect of initial eating disorders on subsequent self-esteem when adjusting for initial self-esteem could be given a similar explanation based on correlations with residuals and regression to the mean.

In a taxonomy of treatment effects (Sorjonen et al., 2024a), the present results do not agree with scenarios with a true decreasing effect of a treatment (e.g., initial self-esteem) on an outcome (e.g., eating disorders). However, the results do agree with a situation where data are generated as in Figure 1, without any direct effects between self-esteem and eating disorders, if we assume an influence by state factors resulting in stronger (more negative) concurrent compared with cross-lagged correlation which, in turn, would result in a positive association between initial self-esteem and subsequent change in eating disorders and vice versa (Equation 2), which is what we found in the present analyses. Actually, in the taxonomy of Sorjonen et al. (2024a), the present results agree with some scenarios where self-esteem would have an increasing, rather than a decreasing, effect on eating disorders and vice versa. However, we prefer the more cautious conclusion that the associations presumably were spurious. Consequently, Krauss et al.s’ suggestion of a vicious circle, where low self-esteem makes people more vulnerable to developing eating disorders and eating disorders, in turn, scars individuals’ self-esteem, may be challenged.

The present findings carry some clinical relevance as they warn against assuming causal effects between low self-esteem and eating pathology. This suggests that measures to improve self-esteem in order to prevent eating pathology, and vice versa, may not be the best use of limited resources. However, our conclusion that self-esteem might not have a decreasing effect on eating pathology is, admittedly, probably of limited practical clinical relevance in itself. But, if challenges of potentially clinically relevant but unsubstantiated claims (e.g., “Wearing an amulet of gold alleviates depression.”) are not allowed, with the argument that the challenges are not clinically relevant (“What is the practical clinical relevance of knowing that wearing an amulet of gold probably does not alleviate depression?”), we risk creating a situation with dire consequences.

The present study is part of a series where we have reanalyzed meta-analyses where researchers have extracted zero-order correlations between constructs measured at two occasions from included studies and estimated adjusted cross-lagged regression effects with Equation 1 (Table 3). Based on our reanalyses, we have concluded that prospective effects were spurious due to correlations with residuals and regression to the mean and, consequently, challenged suggestions of genuine increasing or decreasing prospective effects by authors of the original meta-analyses. This means that the methodological point made in the present paper has been made before, also by others (e.g., Glymour et al., 2005; Eriksson and Häggström, 2014; Castro-Schilo and Grimm, 2018; Lucas, 2023), and that the degree of methodological novelty is limited. However, as researchers continue to overinterpret findings from cross-lagged panel models, despite repeated mentions of biases and flaws in such models, we believe the point is worth repeating. We do not think that it would be tenable to argue that because bias and flaws in adjusted cross-lagged effects, meta-analytically aggregated or not, has been pointed out before, researchers should be allowed to use the method and overinterpret and publish findings without being challenged. Hopefully, we might help spread knowledge that adjusted cross-lagged effects often do not allow causal inference any more than zero-order correlations do.

**Table 3:** Meta-analytic cross-lagged panel analyses, and their conclusions, that we have reanalyzed and challenged.

Challenged study	Challenged presented prospective effect	Challenging reanalysis
Dapp et al. (2023)	Reciprocal between general and domain-specific self-esteem (positive effects)	Sorjonen and Melin (2024b)
Giletta et al. (2021)	Initial peer behavior on subsequent target youth behavior (positive effect)	Sorjonen et al. (2023c)
Harris and Orth (2020)	Reciprocal between self-esteem and quality of social relations (positive effects)	Sorjonen et al. (2023a)
Krauss and Orth (2022)	Reciprocal between self-esteem and work experiences (positive and negative effects)	Sorjonen et al. (2023b)
Prieto-Fidalgo et al. (2022)	Initial mindfulness on subsequent anxiety and depressive symptoms (negative effects)	Sorjonen and Melin (2024a)
Wang et al. (2021)	Reciprocal between social support and posttraumatic stress disorder (negative effects)	Sorjonen and Melin (2023)
Wu et al. (2021)	Reciprocal between academic self-concept and academic achievement (positive effects)	Sorjonen et al. (2024b)

When claiming, as we do here, that other researchers probably have overinterpreted findings due to limitations in used statistical methods, it is common to be asked what statistical methods should have been used instead. This suggests that it is common to believe that inference about true causal effects is possible if using the right statistical method, even with correlational, i.e., non-experimental, data. The random-intercept cross-lagged panel model (RI-CLPM) is an extension, and supposed improvement, of the traditional cross-lagged panel model (Hamaker et al., 2015; Mulder and Hamaker, 2021). Compared with the traditional model, RI-CLPM adjusts for individuals’ trait-like levels on the constructs and estimates auto-regressive and cross-lagged effects between within-individual residuals not accounted for by the trait-like levels. Consequently, effects are estimated within, rather than between, individuals, which presumably allows more confident inference of causality (Usami et al., 2019; Raymaekers et al., 2020; Bailey et al., 2024). However, there are indications that RI-CLPM can be susceptible to bias (Lüdtke and Robitzsch, 2021; Lucas, 2023; Sorjonen et al., 2023d; Murayama and Gfrörer, 2024), meaning that statistically significant effects in RI-CLPM should not be viewed as irrefutable evidence of causality. For example, Sorjonen et al. (2024c) showed that RI-CLPM is susceptible to spurious findings when scores on the two constructs are affected by common auto-correlated state factors, something that might be quite common in psychological research. It should be noted that we could not conduct analyses with RI-CLPM in the present study, as it requires data from at least three waves of measurement. In the present reanalyses we reanalyzed meta-analytic data used by Krauss et al. (2023), which only contained data from two waves of measurement.

Broadly speaking, we do not share the optimism that strong causal inference is possible in analyses of correlational data (see Hammerton and Munafò, 2021; Munafò and Davey Smith, 2018 for a similar opinion). We believe that causal inference is more a question about how data were collected than how they were analyzed. If data were collected with a randomized controlled trial (RCT) with high quality (e.g., adequate sample size and randomization, independent assessments, reliable and valid measures, etc.), a statistically significant treatment condition by time interaction-effect may suggest a causal effect of the treatment. Greater emphasis on experimental designs, such as longitudinal studies with frequent sampling or controlled intervention experiments, could provide more reliable data to investigate causal relationships between self-esteem and eating disorders.

The same confidence about causal inference as in RCTs can probably never be achieved by analyses of correlational, i.e., non-experimental, data. However, with correlational data researchers could, as we did in the present study, analyze models that predict different signs of effects/associations depending on if they are genuinely increasing/decreasing or spurious, i.e., use triangulation (Munafò and Davey Smith, 2018; Hammerton and Munafò, 2021). If findings converge, inference about causality is supported (although never finally proven). If, on the other hand and as in the present study, findings diverge, conclusions about causality would appear premature.

All this said, we still agree with Grosz et al. (2020) that it may be better if researchers make explicit causal claims even when analyzing correlational data, rather than implicit ones in the form of policy recommendations etc., because explicit claims can be challenged. However, this would require a research climate where challenges are welcomed. Unfortunately, today’s scientific atmosphere, including publication and citations, appears to favor statistically significant findings and positive claims (Song et al., 2009; Ioannidis et al., 2014; Antonakis, 2017; Duyx et al., 2017), which means that challenging studies advising caution risk being rejected and ignored. We predict that Krauss et al. (2023) conclusion, that self-esteem and eating disorders reciprocally affect each other, will receive many more citations than our present warning that their findings may have been spurious due to correlations with residuals and regression to the mean.

### Limitations

The present reanalysis suffers from some of the same limitations as the original meta-analysis by Krauss et al. (2023). For example, samples in the included studies were predominantly from Western cultures, of White ethnicity, and of medium to high socioeconomic status. Therefore, it is unclear if and to what degree our main conclusion, that prospective effects between self-esteem and eating disorders appear to be spurious due to correlations with residuals and regression to the mean, applies to other cultural, ethnic, and socioeconomic contexts. A review of literature on eating disorders in different cultural groups showed mixed findings and concluded that it is still unclear if the presentation of eating disorders differs across cultures (Soh et al., 2006).

Used instruments, timing of measurements, the study sample, etc. in the included studies may not always have been optimal. However, it is important to bear in mind that such factors were constant across the analyzed models and cannot, consequently, explain why the models suggested simultaneous and paradoxical decreasing and increasing prospective effects between self-esteem and eating disorders. Therefore, such possible methodological deficiencies should not be a threat against our main conclusion that meta-analytic prospective effects between self-esteem and eating disorders were spurious. We do not think that it would be tenable to argue that due to possible methodological deficiencies in the included studies we should assume genuine causal decreasing reciprocal effects between self-esteem and eating disorders. On the contrary, possible methodological deficiencies should make us even more cautious to assume causality.

We do not claim to have proven, once and for all, that self-esteem and eating pathology have no genuine decreasing effects on each other. The present findings agreed with predictions from a data generating model without any genuine direct effects between self-esteem and eating pathology, like the one in Figure 1, and did not agree with predictions of a hypothesis of genuine decreasing effects. Nevertheless, it is possible that data were generated by some alternative model. However, we do claim that the meta-analytic data analyzed by Krauss et al. (2023) could have been generated by a model without any genuine direct effects between self-esteem and eating pathology, like the one in Figure 1, and that it, therefore, is premature to assume a true decreasing effect between these constructs. We do not think that it would be tenable to argue that because we cannot be absolutely certain that effects between self-esteem and eating disorders are spurious, we should assume that they are genuinely decreasing. It should also be pointed out that, as mentioned above, the meta-analytic data analyzed by Krauss et al., and reanalyzed by us here, actually cannot rule out increasing prospective effects between self-esteem and eating disorders (Sorjonen et al., 2024a).

The present paper was not meant as a comprehensive review of theories, causes, and consequences of self-esteem and eating disorders. Rather, and more specifically, the objective was to reanalyze the meta-analytic data used by Krauss et al. (2023) in order to evaluate if the identified adjusted cross-lagged associations suggested genuine decreasing effects between self-esteem and eating disorders or if the associations may have been spurious due to correlations with residuals and regression to the mean. Readers interested in reviews of research on self-esteem are recommended to read, for example, Leary and MacDonald (2003), Orth and Robins (2014), and Pyszczynski et al. (2004). For reviews of eating disorders, we recommend Schaumberg et al. (2017) and Treasure et al. (2020).

## Conclusion

The present reanalyses of meta-analytic data used by Krauss et al. (2023) indicated that reciprocal prospective decreasing effects between self-esteem and eating disorders appear to have been spurious, presumably due to correlations with residuals and regression to the mean. Consequently, we suggest that the conclusion by Krauss et al. regarding a reciprocal-relations model of low self-esteem and eating disorders is not supported. It is important for researchers not to overinterpret findings when analyzing correlational, i.e., non-experimental, data. Neither should results from meta-analyses be taken at face value, as they may be nothing more than aggregations of biased findings. Our results suggest caution in interpreting the prospective effects between self-esteem and eating disorders. However, future studies with more robust methodological approaches may better clarify these dynamics.

## Data Availability

Publicly available datasets were analyzed in this study. This data can be found here: https://osf.io/g8dkc/.
